# Interobserver agreement and clinical disparity between the Graf method and femoral head coverage measurement in developmental dysplasia of the hip screening: A prospective observational study of 198 newborns

**DOI:** 10.1097/MD.0000000000026291

**Published:** 2021-06-18

**Authors:** Wen-Chieh Chang, Kuei-Hsiang Hsu, I.-Fang Lo, Kai-Hao Liao, Yu-Ping Su

**Affiliations:** a Department of Orthopaedics and Traumatology, Taipei Veterans General Hospital; b Department of Orthopaedics, School of Medicine, National Yang Ming Chiao Tung University; c Department of Nursing, Taipei Veterans General Hospital, Taipei, Taiwan.

**Keywords:** developmental dysplasia of the hip, femoral head coverage, Graf method, hip ultrasound, interobserver agreement

## Abstract

Ultrasonography is the ideal tool for assessing hip morphology in infants younger than 6-month-old. This study assessed the interobserver agreement and clinical disparities of the 2 most widely used ultrasound (US) methods, the Graf method, and femoral head coverage (FHC) measurement.

A prospective observational study (STROBE compliant) of 2024 newborns was conducted between January 2017 and December 2018. Hip US was conducted on all newborns with abnormal Barlow and Ortolani maneuvers as well as on 50 randomly selected normal newborns. The physical examination and US were performed by a senior pediatric orthopedic surgeon with musculoskeletal sonography certification. Three observers with different levels of experience interpreted the images by using the Graf method and FHC. We analyzed the intraclass correlation coefficient, Cohen kappa, and the disparity between the clinical findings of the 2 methods.

A total of 198 newborns (9.8%) presented with clinical instability, including 193 subluxatable hips in 168 patients (84.8%) and 45 dislocatable/dislocated hips in 30 patients (15.2%). The mean age at US examination was 11.69 days (range: 0–18 days). The intraclass correlation coefficient was .71 (95% CI: 0.55–0.83) for FHC, 0.63 (95% CI: 0.38–0.78) for the alpha angle, and 0.47 (95% CI: 0.16–0.69) for beta angle. The Cohen kappa coefficients of Graf type were 0.19 (95% CI: 0.03–0.35), 0.39 (95% CI: 0.20–0.58), and 0.17 (95% CI: 0.02–0.32) between observers 1 and 2, observers 1 and 3, and observers 2 and 3, respectively. Based on the Graf method, 14% of the stable hips had abnormal USs; by contrast, 19.2% of the subluxatable hips and 17.8% of the dislocatable/dislocated hips had normal Graf morphologies. In USs interpreted using FHC, 16% of stable hips demonstrated abnormal coverage, whereas 13.5% of subluxatable hips and 4.4% of dislocatable/dislocated hips had normal FHC.

Incidence of clinically detectable hip instability was 9.8% among newborns in our series. Both alpha angle and FHC ratio revealed substantial interobserver agreement while beta angle achieved moderate agreement. FHC ratio possesses higher sensitivity and similar specificity compared with the Graf method when screening unstable hips.

Level II, development of diagnostic criteria on basis of consecutive patients

## 1 Introduction

Developmental dysplasia of the hip (DDH) presents with a spectrum of diseases ranging from mild dysplasia to hip dislocation. Clinically detectable hip instability has a prevalence ranging from 1 to 28 per 1000 infants.^[1,2]^ Universal physical examination followed by selective hip ultrasound (US) is generally used as the standard screening strategy.^[2,3]^ According to the *2015 American Academy of Orthopaedic Surgeons Guideline on Detection and Nonoperative Management of Pediatric Developmental Dysplasia of the Hip in Infants up to Six Months of Age*, moderate evidence supports performing an imaging study for infants with the following risk factors: breech presentation, family history, and history of clinical instability.^[2]^

Sonographic findings are suggested to be prognostic. Some studies have shown that patients with poor sonography measurement presented with a higher degree of instability, and responded less effectively to harness wearing and bracing.^[4–6]^

The consistency of US results between observers with different levels of experience has been questioned. According to studies, the interpretation for the same US image varies.^[7–12]^ In addition to the interobserver agreement, the reliability of sonography remains questionable. Many studies have reported that US images may not always be compatible with clinical findings; some unstable hips may exhibit normal hip morphology, whereas some stable hips may present Graf III or IV dysplasia.^[13–17]^

Currently, the primary methods for interpreting US findings are Graf method and the percentage of femoral head coverage (FHC); they both provide simple and quantitative results for proper diagnosis.^[18,19]^ Although the alpha angle and FHC have been reported to be positively correlated,^[20]^ firm conclusions on their consistency and clinical efficacy are difficult to draw from studies.^[18]^ In many studies, image acquisition and interpretation, as well as physical examination, were performed by different specialists, which may have increased the discrepancy.^[13–17]^

The primary purpose of this study was to compare the interobserver agreement of the Graf method and FHC ratio for independently acquired sonographic images; the secondary purpose was to investigate their correlation with different levels of hip stability.

## 2 Materials and methods

We conducted a prospective observational study (STROBE compliant) from January 2017 to December 2018. During this time period, all newborns delivered in *Taipei Veterans General Hospital, Taipei, Taiwan,* or referred for hip checkups were included. Baseline data including gestational age, birth weight, Apgar score, and presence of risk factors for DDH (including female sex, firstborn, twins, and breech presentation) were recorded from the medical chart. This study was approved by the institutional review board (IRB) of the authors’ hospital (IRB number 2019–06-010AC, Jun 18, 2019). Exclusion criteria included premature newborns, syndromic and neurogenic dislocation.

A senior pediatric orthopedic surgeon with musculoskeletal sonography certification performed general surveys, hip physical examinations, and selective hip sonography for the included newborns. The Barlow and Ortolani tests were performed while the newborn in supine position, the hips were positioned in neutral rotation and 90-degree flexion. To perform the Barlow test, the examiner adducted the hip joint while applying a posteriorly directed force on the knee to provoke dislocation; the Ortolani test was examined by abducting the hip joint while applying an anteriorly directed force on the femur to reduce the dislocated hip joint. Hip stability was classified according to the Barlow and Ortolani test into 3 groups: normal, subluxatable, and dislocatable or dislocated. Normal stability was defined as the hip center remaining static during the Barlow and Ortolani maneuver; subluxatable hips were defined as any movement of the hip center without dislocation during the examination; dislocatable or dislocated hips were defined as the hip center completely displacing from the acetabulum during the test. Confirmation of a subluxatable hip required the agreement of 2 senior pediatric orthopedic surgeons.

**Figure 1:**
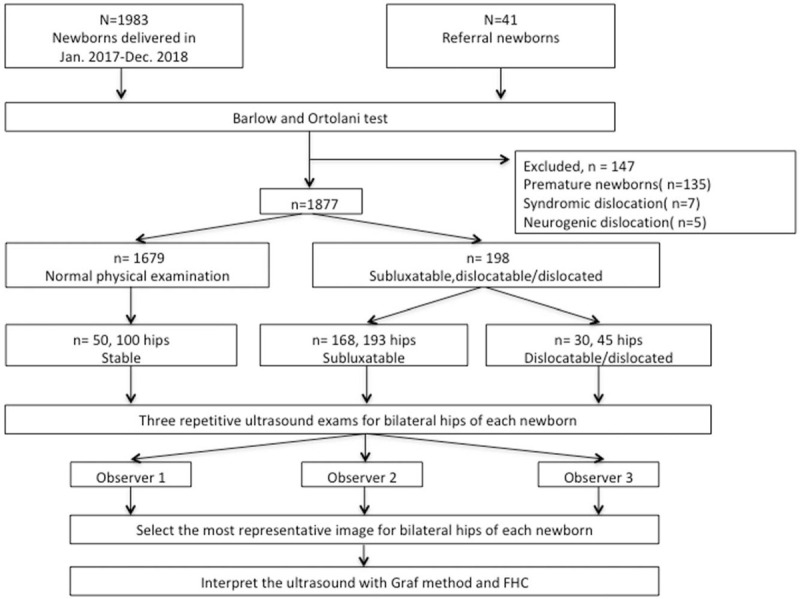
Flowchart of inclusion and exclusion of patients in this study. FHC: femoral head coverage.

After physical examination, newborns with subluxatable, dislocatable, or dislocated hip received three repetitive static US examinations for bilateral hips after receiving the consent of their parents. To obtain the ultrasonic measurements of clinically stable hips, 50 newborns with normal physical examinations were randomly selected for three repetitive US examinations on bilateral hips (Fig. 1). The minimal effective sample size was calculated based on power analysis exceeded 80%

A proper US image should present the lower iliac margin at the triradiate cartilage, the chondroosseous border of the proximal femur, the labrum, and the deepest point of the acetabulum.^[21]^ The most representative image from bilateral hips of examined newborns was independently selected by 3 observers with different levels of practice, including a senior attending physician, a fellowship-trained surgeon, and a medical student. After the most representative image was selected, the alpha angle, beta angle, Graf type and FHC ratio of the hip were independently interpreted by three observers (Fig. 1). All observers were blinded to the patients’ profiles, the clinical findings, and the sonographic interpretations of other observers.

**Figure 2:**
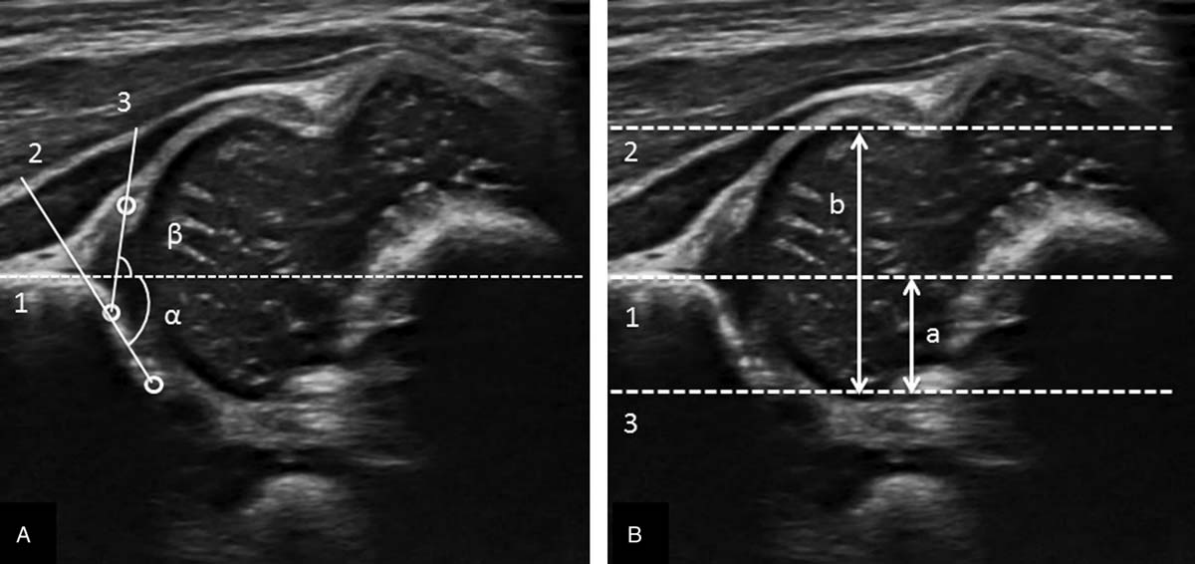
(A) Graf method. 1. Base line. 2. Bony roof line. 3. Cartilaginous roof line. (B) Femoral head coverage ratio; calculated as a/b. 1. Base line. 2. Line parallel to base line and connected to the most lateral femoral head. 3. Line parallel to base line and connected to the most medial femoral head.

The 3 observers agreed on both techniques before interpretation (Fig. 2). The alpha and beta angle, described by Graf in 1980,^[22]^ were formed by the vertical cortex of the ilium (base line) and the bony acetabular roof line, the base line and the triangular labral fibrocartilage line, respectively. According to Graf classification, type I was defined as alpha angle greater or equal to 60 degrees; type IIa and IIb were defined as alpha angle between 50 and 59 degrees in newborn younger or older than age of 3 months, respectively; type IIc and type D were defined as alpha angle between 43 to 49 degree with beta angle less or greater than 77 degrees, respectively; type III and IV were dislocated hip defined as alpha angle less than 43 degrees with absence or presence of inverted labrum, respectively. Graf type >I was defined as immature.^[22]^ The FHC ratio was calculated as the distance between the base line and the parallel line connected to the most medial femoral head divided by the distance between the two lines parallel to base line and connected to the most lateral and medial femoral head.^[19,23]^. FHC < 50% was defined as abnormal.^[18–20]^

The Graf type and FHC ratio from the most senior observer were further grouped into three categories according to the initial Barlow and Ortolani test result: normal, subluxatable, dislocatable, or dislocated. Using the physical examination finding as standard reference, the sensitivity and specificity of Graf type and FHC ratio to each level of hip stability were calculated.

The primary outcome variables were the intraclass correlation coefficients (ICC) of alpha angle, beta angle, Graf type and FHC ratio among 3 different observers; the secondary outcome variables were the sensitivity and specificity of FHC ratio and Graf type by using the Barlow and Ortolani test as the standard reference. In the current study, all USs were performed by an experienced senior pediatric orthopedist to ensure consistency in quality. Three images for each hip were provided for observers to self-align, thus minimizing intraobserver bias.

The ultrasonography device was equipped with a 7.5-MHz linear transducer (LOGIQ e ultrasound, GE Healthcare, USA). Infants were placed in the lateral decubitus position while US examination. The transducer was placed vertically on the hip joint.^[21]^

### 2.1 Statistical analysis

Pearson chi-square test was used to compare categorical variables. A one-way analysis of variance was used to analyze the association of the alpha angle, beta angle, and FHC among normal, subluxatable, and dislocatable/dislocated hips; Tukey's method was used for post hoc testing. The missing data were excluded for statistical analysis.

The intraclass correlation coefficient (ICC) was used to evaluate the agreement of alpha angle, beta angle, and FHC ratio between observers. Cohen kappa test was used to investigate the reliability of Graf typing between observers. ICC or kappa values less than 0 indicated poor agreement, whereas values of 0 to 0.20, 0.21 to 0.40, 0.41 to 0.60, 0.61 to 0.80, and 0.81 to 1.0 indicated slight, fair, moderate, substantial, and excellent agreement, respectively. Considering the initial Barlow and Ortolani test results (normal, subluxatable, dislocatable, or dislocated) as the standard reference, the sensitivity and specificity of Graf type and FHC ratio to different levels of hip stability were calculated and presented with 95% confidence interval. Significance was defined as *P* < .05. Calculations were performed using SPSS version 22 (SPSS, Armonk, NY).

## 3 Results

From January 2017 to December 2018, 2024 newborns underwent general hip physical examinations (Fig. 1), 41 of whom were referred for further DDH evaluation. A total of 147 newborns were excluded because of prematurity (n = 135), syndromic dislocation (n = 7), or neurogenic dislocation (n = 5). Moreover, 1679 newborns (82.9%) had normal clinical findings. A total of 198 newborns (9.8%) presented with clinical instability and underwent subsequent ultrasound examination; these included 193 subluxatable hips in 168 patients (84.8%) and 45 dislocatable/dislocated hips in 30 patients (15.2%). The baseline demographic data for the groups are summarized in Table 1. No statistical difference was observed among the groups in terms of gestational age (in weeks, *P* = .75), Apgar score (*P* = .53), birth weight (*P* = .06), sex (*P* = .51), twin status (*P* = .60), first born status (*P* = .72), or breech presentation (*P* = .54).

**Table 1:** Patient characteristics.

	Normal	Subluxatable	Dislocatable/dislocated	*P value*
Patient numbers	50	168	30	–
Mean age at US (days)	13.23 (0–18)	11.34 (0–15)	11.12 (0–13)	.54
GA (weeks)	38.18 (37–41)	38.25 (37–41)	38.44 (37–40)	.75
Apgar score	7.77 (6–8)	7.72 (6–8)	7.71 (6–8)	.53
Birth weight (g)	2998.54 (2282–4218)	2950.57 (2352–4058)	2815.29 (2426–3780)	.06
Female	33 (66%)	97 (57.7%)	17 (56.7%)	.51
Twins	3 (6%)	7 (4.2%)	3 (10%)	.60
First born	21 (42%)	65 (38.7%)	12 (40%)	.72
Breech	12 (24%)	17 (10.1%)	6 (20%)	.54

The interobserver analysis of the total 496 hips from 248 enrolled newborns were reviewed by 3 observers (Fig. 1), revealing substantial agreement regarding the alpha angle (ICC: 0.63, 95% CI: 0.38-0.78) and FHC (ICC: 0.71, 95% CI: 0.55–0.83) interpretations (Table 2); however, the ICC was only moderate for the beta angle (ICC: 0.47, 95% CI: 0.16–0.69). Agreement for the Graf type ranged from slight to fair. The kappa coefficients were .19 (95% CI: 0.03–0.35), 0.39 (95% CI: 0.20–0.58), and 0.17 (95% CI: 0.02–0.32) for observers 1 and 2, observers 1 and 3, and observers 2 and 3, respectively.

**Table 2:** Interobserver reliability for sonogram interpretation.

US parameter (n = 496 hips)	ICC	95%CI
Alpha angle	0.63	0.38–0.78
Beta angle	0.47	0.16–0.69
FHC	0.71	0.55–0.83

Graf type (n = 496 hips)	*κ* value	95%CI
Observer 1–Observer 2	0.19	0.03–0.35
Observer 1–Observer 3	0.39	0.20–0.58
Observer 2–Observer 3	0.17	0.02–0.32

The mean alpha angles for stable, subluxatable, and dislocatable/dislocated hips (Table 3) were 65.74° (range: 48.79°–79.57°), 54.03° (range: 35.25°–60.65°), and 49.17° (range: 25.19°–65.71°), respectively, and the mean FHC values were 55.08% (range: 43%–70%), 44.94% (range: 38%–56%), and 36.84% (range: 21%–53%), respectively. Significant differences (*P* < .001) were observed between groups in the post hoc test for alpha angle and FHC.

**Table 3:** Distribution of ultrasound measurement for different levels of instability.

US parameter	Stable (100 hips)	Subluxatable (193 hips)	Dislocatable/dislocated (45 hips)	*P* value
Mean α angle (range)	65.74 (48.79–79.57)	54.03 (35.25–60.65)	49.17 (25.19–65.71)	<.001
Mean FHC ratio (range)	55.08 (43–70)	44.94 (38–56)	36.84 (21–53)	<.001

Discrepancies between US and clinical findings are shown in Table 4. When using Graf method, 14% of the clinically stable hips had abnormal USs; moreover, 19.2% of subluxatable hips and 17.8% of dislocatable/dislocated hips had normal Graf morphologies. Among the 100 stable hips, 86 were Graf type I, 13 were type IIa, and 1 was type IIc; among the 193 subluxatable hips, 37 (19.2%), 113 (58.5%), 13 (6.7%), 11 (5.7%), 17 (8.8%), and 2 (1%) were Graf type I, IIa, IIc, D, III, and IV, respectively; of the 45 dislocatable/dislocated hips, 8 (17.8%), 6 (13.3%), 2 (4.4%), 8 (17.8%), 10 (22.2%), and 11 (24.4%) were Graf type I, IIa, IIc, D, III, and IV, respectively. By using FHC, 16% of stable hips demonstrated abnormal coverage, whereas 13.5% of subluxatable hips and 4.4% of dislocatable/dislocated hips had normal FHC.

**Table 4:** Association of Graf method and femoral head coverage with instability groups.

US method^∗^	Stable (100 hips)	Subluxatable (193 hips)	Dislocatable/dislocated (45 hips)
Graf type I	86 (86%)	37 (19.2%)	8 (17.8%)
Graf type IIa	13 (13%)	113 (58.5%)	6 (13.3%)
Graf type IIb	0	0	0
Graf type IIc	1 (1%)	13 (6.7%)	2 (4.4%)
Graf type D	0	11 (5.7%)	8 (17.8%)
Graf type III	0	17 (8.8%)	10 (22.2%)
Graf type IV	0	2 (1%)	11 (24.4%)
FHC ≥ 50%	84 (84%)	26 (13.5%)	2 (4.4%)
FHC < 50%	16 (16%)	167 (86.5%)	43 (95.6%)

The sensitivity and specificity of ultrasonography for the detection of unstable hips were analyzed using clinical findings as a reference. In the subluxatable group, the sensitivity and specificity were 80.83% (95% CI: 74.56%–86.13%) and 86.0% (95% CI: 77.63%–92.13%), respectively, for the Graf method and 83.42% (95% CI: 77.41%–88.37%), and 84.0% (95% CI: 75.32%–90.57%), respectively, for the FHC method. In the dislocatable/dislocated group, the sensitivity and specificity were 82.22% (95% CI: 67.95%–92.0%) and 86.0% (95% CI: 77.63%–92.13%), respectively, for the Graf method and 95.56% (95% CI: 84.85%–99.46%) and 84.0% (95% CI: 75.32%–90.57%), respectively, for the FHC method.

## 4 Discussion

In the present study, the interobserver study demonstrated substantial agreement on the FHC and alpha angle with an ICC of 0.71 and 0.63, respectively, and only moderate agreement on the beta angle with an ICC of .47. In hips with normal FHC ratio (FHC ≥ 50%), 13.5% were subluxatable and 4.4% were dislocatable/dislocated hips; in normal Graf morphology (Graf type I), more subluxatable (19.2%) and dislocatable/dislocated (17.8%) hips were observed. The specificity was similar in both methods (Graf: 86%, FHC: 84%), and the sensitivities of FHC for subluxatable and dislocatable/dislocated hips were 83.43% and 95.56%, respectively. The FHC results were superior to those of the Graf method, which had sensitivities of 80.83% for subluxatable hips and 82.22% for dislocatable/dislocated hips.

Numerous studies have reported that, when using the Graf method, measurements of the alpha angle are more consistent than those of the beta angle.^[8,9,11]^ In a study of 66 scans, Copuroglu et al^[11]^ reported ICC values of .72 for the alpha angles of both hips, 0.47 for the beta angle of the right hip, and .63 for the beta angle of the left hip among 7 observers. In a test of 20 US images acquired by a single radiologist and interpreted by 22 orthopedic surgeons of different levels of practice, Omeroglu et al^[8]^ reported average interobserver differences for the alpha and beta angles of 5.1° and 10.1°, respectively; the intraobserver and interobserver agreement ratios for Graf types were 65% and 51%, respectively. In a study on agreement among a radiology team, an orthopedist, and a pediatrician, Simon et al^[9]^ obtained a higher ICC for the alpha than for the beta angle, and the highest agreement of 90% existed between the orthopedist and pediatrician. In a study of 2071 scans obtained from a single sonographic operator, Pedrotti et al^[12]^ stated that the ICC was greater than 0.80 for both the alpha and beta angles between the operator and another external reader.

FHC is positively correlated with the alpha angle,^[20]^ and it reflects the acetabular index.^[23]^ Moreover, the FHC has prognostic value for dislocatable hips when treated using a Pavlik harness.^[4,5]^ However, no solid evidence supports the superiority of the FHC method for clinical use. In a series comparing the Graf and FHC methods in 657 newborns, Czubak et al reported good agreement between 2 orthopedic surgeons with US experience and 2 students when using the FHC method. Although both methods have similar results for detecting dislocated or subluxated hips, the FHC method has higher specificity and interobserver agreement.^[7]^ Falliner et al evaluated 232 newborns by using the Graf and FHC methods; the ICC of the alpha angle ranged from 0.72 to 0.74, and that of the FHC ranged from 0.61 to 0.77. Notably, no clear difference was observed between 5 experienced physicians and 5 students.^[10]^ A similar phenomenon was observed when using plain film to assess reduction quality in DDH patients. In a series of 28 patients interpreted with post-operation plain film using the MRI as standard reference, Yong et al reported no significant difference in rating reduction quality between different levels of experience and specialties.^[24]^

To the best of our knowledge, no consensus has been reached regarding the disparity between ultrasonography and clinical manifestation. In some series, examiners with different specialties and experience may have increased this discrepancy. Tönnis et al^[13]^ reported that nearly half of Graf pathological hips had no signs of instability after screening 1310 newborns. Malkawi et al^[14]^ found that only 21.9% of dislocated hips were compatible with the clinical diagnosis in a series of 4438 newborns. Dogruel et al^[16]^ reported that only 13.7% of clinically pathological hips had Graf abnormalities in 3541 infants. Arti^[15]^ performed the Graf method and the Barlow and Ortolani test for 11402 hips and stated that 8.6% of clinically unstable hips had normal hip USs. Kyung et al^[17]^ performed clinical hip screenings for 2686 infants; 92.7% of the subluxatable hips and 73.7% of the dislocatable hips were Graf type I or IIa.

The lower reliability of the beta angle in our series was attributed to inconsistency in locating the correct landmark at the transitional point from the concavity to the convexity of the iliac bony rim. Additionally, the center point of the triangular labral fibrocartilage could not be precisely located in a small percentage of the USs. The good interobserver agreement is reasonable to assume for FHC. The iliac bony rim and the medial and lateral parts of the femoral head for FHC measurement are easier to locate than are the landmarks used in the Graf method.

Several reasons may explain the disparity between the US image and clinical finding. First, newborns may not have been relaxed during physical examinations, and tension in the soft tissue and muscle tone may have affected hip stability. Second, although USs and physical examinations were performed by experienced pediatric orthopedic surgeons, the image may not have correlated perfectly with the clinical findings; such a discrepancy could be reduced but never eliminated.

The main strength of this study is that it provided a comprehensive comparison of interobserver agreement, sensitivity, and specificity between the two most widely used US methods on detecting the unstable hips, which was not clearly mentioned in previous works of literature, our results may aid to improve the screening strategy of developmental dysplasia of hip. This study had several limitations. Intraobserver variance may have affected the results of US interpretation; the sample size was relatively small; a comprehensive dynamic US was not performed; and long-term follow-up data were lacking. Although inferior ultrasonography is associated with treatment failure in dislocatable/dislocated hips,^[4–6]^ whether ultrasonography has a prognostic role in mildly unstable hips may require further investigation.

In this study, clinical assessment is still recognized as the first line for hip stability in newborn screening, however, we suggest initiate US examination if the presence of any suspicious physical examination. Ultrasound can provide an objective result for continuous assessment on severity stratification and treatment efficacy.

In conclusion, the incidence of clinically detectable hip instability was 9.8% among newborns in our series. Both alpha angle and FHC ratio revealed substantial interobserver agreement while beta angle achieved moderate agreement. FHC ratio possesses higher sensitivity and similar specificity compared with the Graf method when screening unstable hips.

## Acknowledgments

The authors acknowledge the biostatistics center of our institute for statistical analyses and consultations.

## Author contributions

**Conceptualization:** Yu-ping Su.

**Data curation:** I-Fang Lo, Kai-Hao Liao.

**Formal analysis:** Kuei-Hsiang Hsu.

**Investigation:** Wen-Chieh Chang, Kuei-Hsiang Hsu, Kai-Hao Liao, Yu-ping Su.

**Supervision:** Yu-ping Su.

**Writing – original draft:** Wen-Chieh Chang.

**Writing – review & editing:** Yu-ping Su.
